# Supplementary material for: Bias in Universal Machine-Learned Interatomic Potentials and Its Effects on Fine-Tuning
Source: J Chem Theory Comput. 2026 Jun 20;22(13):6820–34. doi: 10.1021/acs.jctc.6c00425 (PMC13379080; doi:10.1021/acs.jctc.6c00425)
Supplement: Supplementary file 1 [file ct6c00425_si_001.pdf]

Supporting Information for Bias in Universal Machine-Learned Interatomic Potentials and its Effects on Fine-Tuning

Nicolas H. Wong[a] and Julia H. Yang[a*]

[a] *Department of Chemical Engineering, Georgia Institute of Technology, Atlanta, GA 30363, United States*

E-mail: jhyang@gatech.edu

Supplemental Information

Linear Response Theory

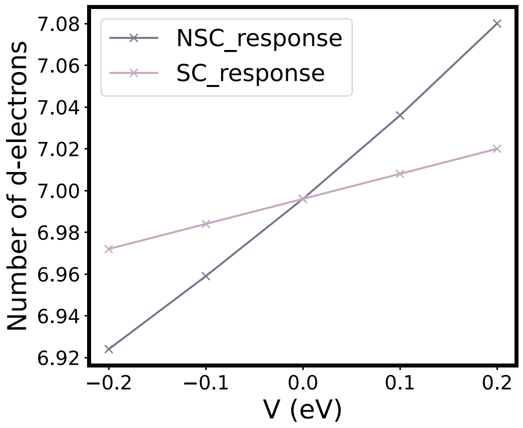

Figure S1: Linear response theory for $CoCl_2$. Self-consistent response slope = 0.120, Non self-consistent response slope = 0.389.

The Hubbard U correction is defined as:[1]

$$U = \chi^{-1} - \chi_0^{-1} \approx \left(\frac{\partial N_I^{SCF}}{\partial V_I}\right)^{-1} - \left(\frac{\partial N_I^{NSCF}}{\partial V_I}\right)^{-1} = \frac{1}{0.12} - \frac{1}{0.389} \approx 5.76\ eV$$

The Hubbard U correction selected for cobalt is 5.76 eV, which is relatively high compared to literature. Literature on different metal-ligand bonds, namely oxides, crystalline materials, find a correction of 3.32 eV.[2,3] As we are consistently fitting to this data, this should not have a significant effect on evaluations.

Iterative Training

We plot the difference of each training iteration from DFT. Figure S2 displays the results from iterative data generation, where we plot each model's energy evaluation against DFT as models are trained.

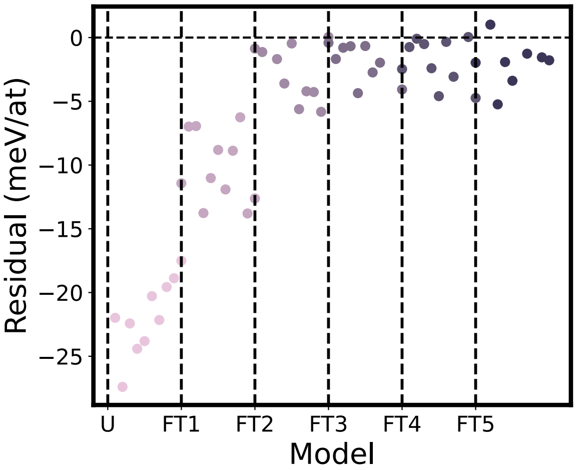

Figure S2: Training errors from iterative dataset generation as residuals against DFT. Each new model is trained on all of the preceding data. The y-axis represents the residual as MD against DFT (E_{MD}-E_{DFT}). The x-axis represents the model used to generate the trajectory, with colors to indicate different models. Lighter colors are earlier models and darker colors are later models.

We observe a plateau in residuals after FT2 during training, where models average an RMSE of 5 meV/at.

Noniterative Workflow

We use the noniterative analysis as a control test against the iterative workflow. Here, we generate a workflow that only uses data from the universal potential, using one sampled trajectory. Figure S3 illustrates this workflow.

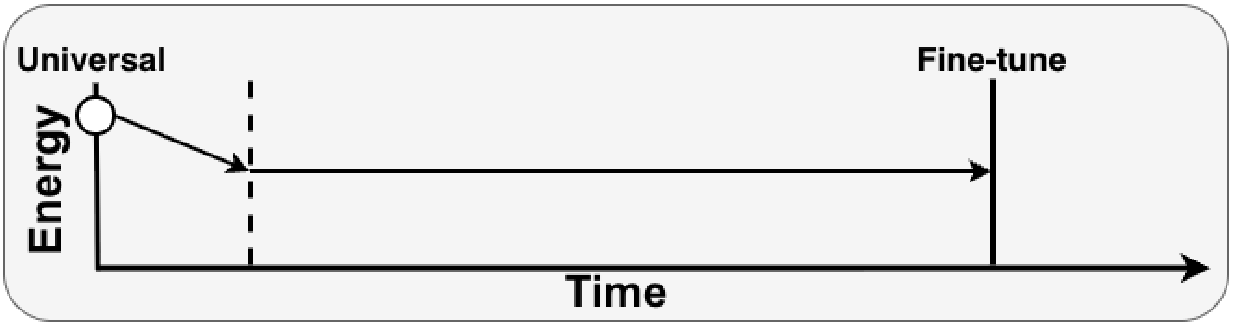

Figure S3: Noniterative dataset data generation strategy. The circle denotes a starting configuration, and the arrows represent MD trajectories, with equilibration and production runs separated by dashed lines. This workflow uses a universal potential to sample a long trajectory and fine-tunes on the results of the trajectory.

For non-iterative fine-tuning (NI-Xpts), shown in Figure S3, we only initialize one starting configuration, and sample MD until X DFT points converge, where X is chosen to match the number of data points to the iterative fine-tuning workflow, resulting in the following models: NI-10pts, NI-21pts, NI-31pts, NI-40pts, NI-50pts.

We perform test evaluations against a test set unseen by each model (N=22). Figure S4 visualizes potential energy data distribution both of the respective fine-tuned model and the test set, and the average error on predictions.

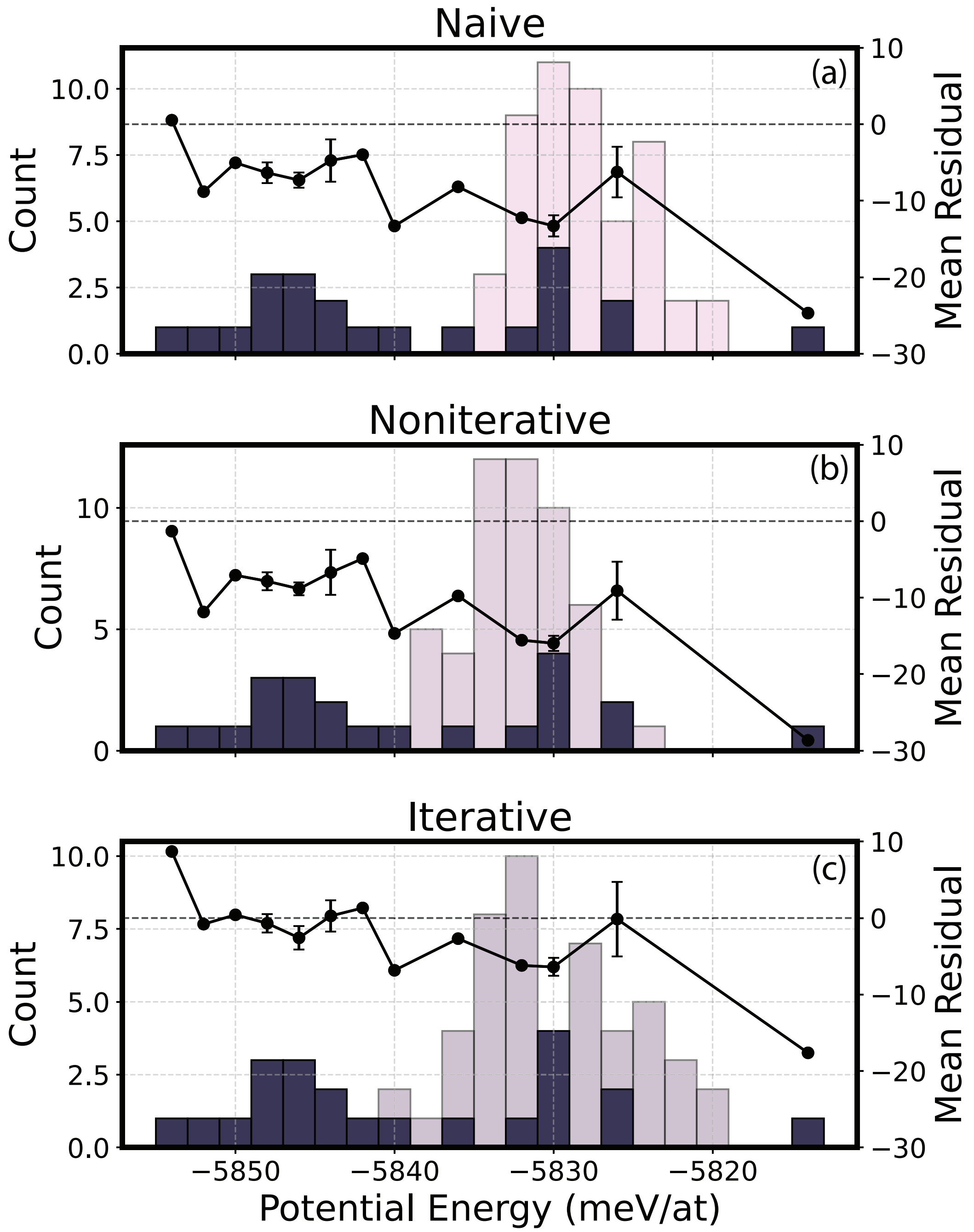

Figure S4: Model errors against a randomly generated test-set. Each panel describes the distribution of potential energies that each model was fine-tuned with in lighter colors, and the test set that each model was evaluated against in a darker color. On the secondary axis, the average residual of the respective energy. The top panel (a) describes results for the naive fine-tuning approach, the middle panel (b) describes results for the noniterative approach, and the bottom panel (c) describes the results for the iterative fine-tuning approach. Error bars represent prediction standard deviation within each histogram bin.

Interestingly, each model appears to have similar trends in fitting the dataset, with similar shapes between each line. However, iterative models perform the best across the board, with minimal error for most structures. Table 1 summarizes Figure S4 using RMSEs.

Table S1: Noniterative Model Metrics on Independent Test Set, N=22

**Metric**	$RMSE_E$ meV/at	$RMSE_{F_x}$ eV/Å	$RMSE_{F_y}$ eV/Å	$RMSE_{F_z}$ eV/Å	$RMSE_S$ eV/Å^3
**Universal Model**					
U	21.24	0.223	0.221	0.229	0.0019
**NI-X Models**					
NI-10pts	15.31	0.202	0.206	0.215	0.0024
NI-21pts	13.48	0.198	0.202	0.211	0.0016
NI-31pts	11.98	0.194	0.197	0.207	0.0016
NI-40pts	**11.70**	0.192	0.195	0.206	0.0014
NI-50pts	12.32	**0.191**	**0.194**	**0.204**	**0.0012**

Noniterative models perform worse significantly overall against iterative (Best: 5.79 meV/at) and naive models (Best: 9.02 meV/at), indicating that their fine-tuned domain is smaller than the other models, limiting their reliability across the domain of the same chemistry. Figure S5 illustrates the performance of each model on sampling MD, as compared to the other two workflows.

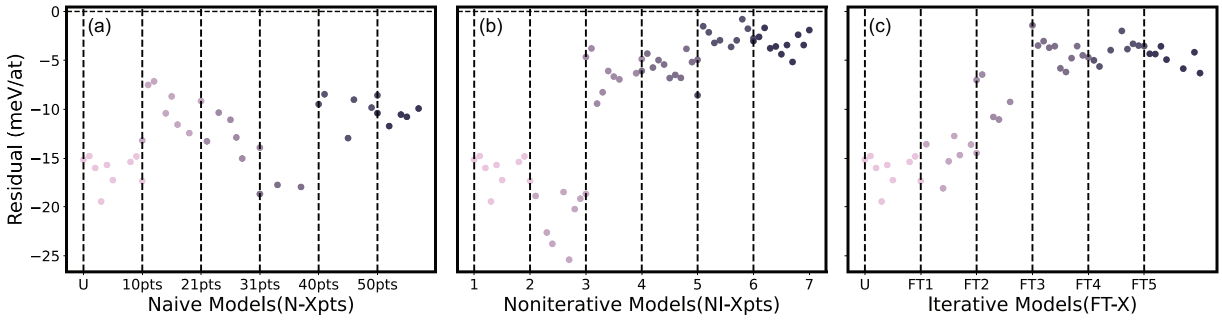

Figure S5: Testing errors from evaluations as residuals against DFT. The y-axis represents the residual as MD against DFT (E_{MD}-E_{DFT}). For each workflow, the x-axis represents the model used to generate the trajectory, with colors to indicate different models. Lighter colors are earlier models and darker colors are later models. The left (a) panel represents the naive workflow, the middle (b) panel represents the noniterative workflow, and the right (c) panel represents the iterative workflow.

Noniterative models appear to perform the best here, but when combined with the knowl-

edge of Table S1, we suggest that this workflow overfits models. When evaluated on new structures, the noniterative models fail, suggesting that these results will not be consistent across geometries. We summarize these results in Table S2.

Table S2: Model Metrics on Self-Generated MD Test Set

**Metric**	$\mathrm{RMSE_E}$ meV/at	RMSE_{F_x} eV/Å	RMSE_{F_y} eV/Å	RMSE_{F_z} eV/Å	$\mathrm{RMSE_S}$ eV/Å^3	N
**Universal Model**						
U	16.28	0.175	0.157	0.182	0.0024	9
**NI-X Models**						
NI-10pts	21.05	0.151	0.156	0.152	0.0012	8
NI-21pts	6.56	0.143	0.138	0.135	**0.0009**	9
NI-31pts	5.59	0.139	0.124	0.122	0.0012	11
NI-40pts	3.64	0.109	0.111	**0.097**	0.0014	10
NI-50pts	**3.37**	**0.103**	**0.108**	0.109	0.0013	11

Figure S6 describes the data coverage of each workflow as compared to each other.

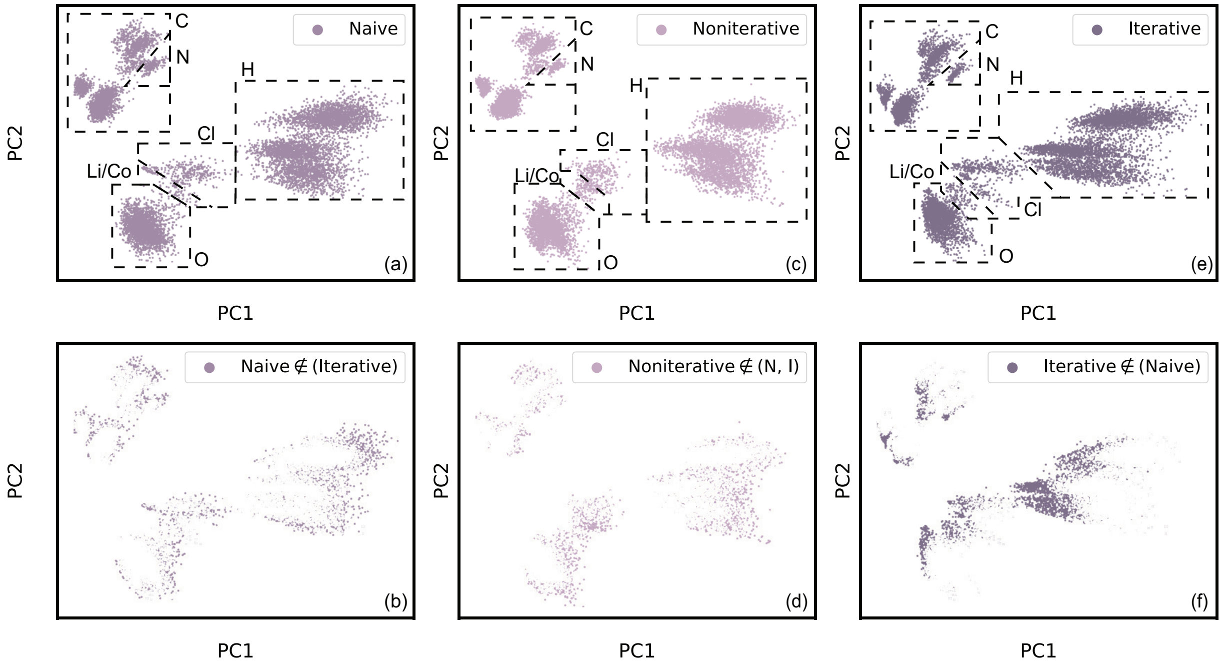

Figure S6: Principal component analysis of the final 50 point datasets, with regions labeled by atom type. The first two principal components account for 42.27% of explained variance. Panels (a), (c) and (e) show the full datasets, while panels (b), (d) and (f) display only the unique regions sampled by each method, which is represented visually by overlaying white coloring on top of the corresponding dataset, constituting for example, Naive∉Iterative. The left panels (a, b) correspond to the naive workflow, the middle panels (c, d) correspond to the noniterative workflow, and the right panels (e, f) correspond to the iterative workflow.

Upon visual inspection, the dataset for the noniterative workflow is very similar to that of the naive workflow. We further decompose these datasets per iteration in Figure S7.

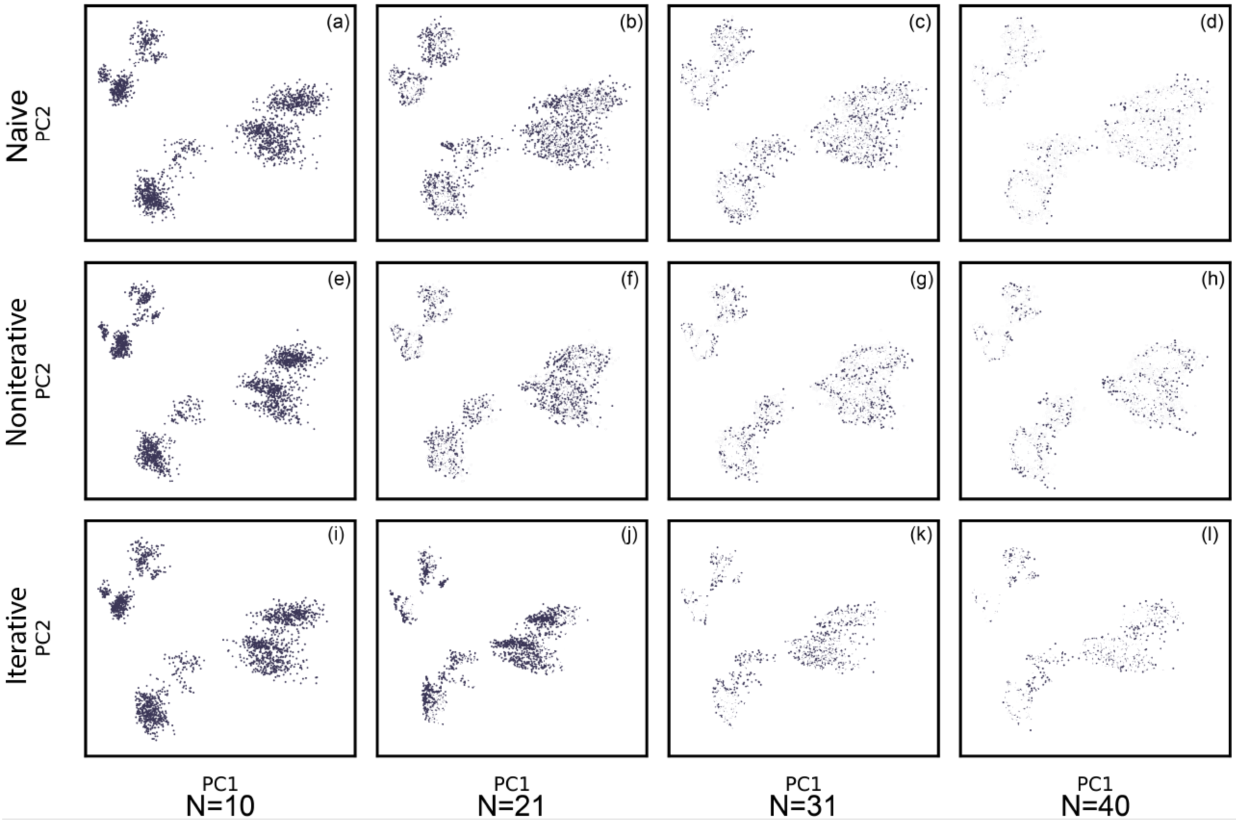

Figure S7: Principal component analysis of each dataset from the naive, noniterative, and noniterative approaches. Each panel represents the unique coverage contributed by the most current iteration (left to right); coverage of previous iterations is overlayed in white. Panels (a-d) correspond to datasets used to generate N-10, N-21, N-31, and N-40 respectively. Panels (e-h) and (i-l) likewise correspond to datasets used to generate NI-10 and FT-1, NI-21 and FT-2, etc., respectively.

Compared to the other two workflows, the noniterative workflow is very similar to the naive workflow, where data expands outwards.

Bond Length Histograms

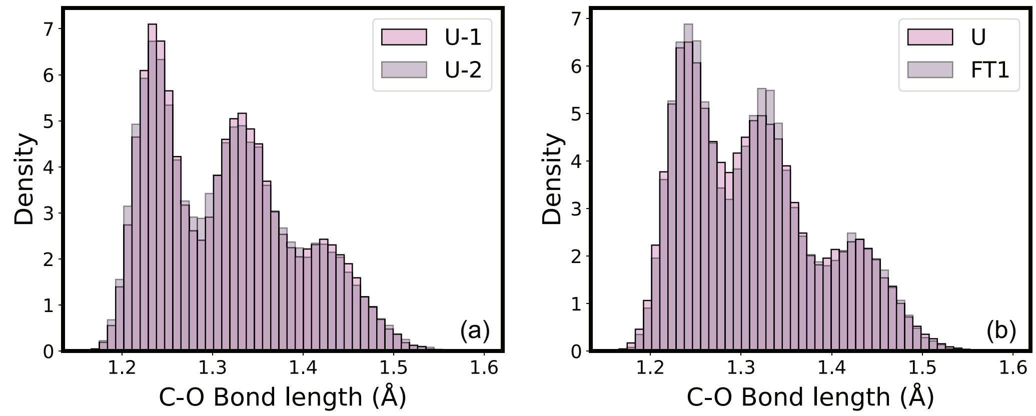

Figure S8: A histogram of C−O bond lengths seen in universal potentials (a) and fine-tuned potentials (b). Panel (a) depicts C−O bond lengths from two trajectories generated by a universal potential. Panel (b) depicts C−O bond lengths from a universal potential (U) and a fine-tuned potential (FT1)

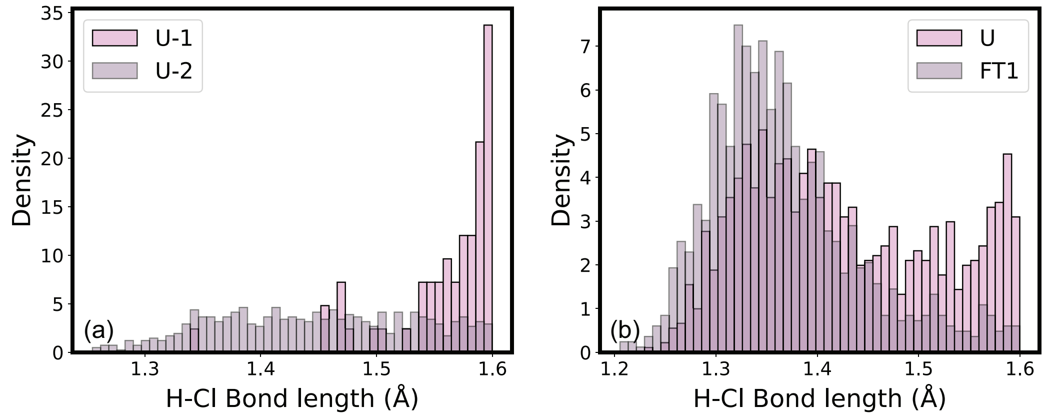

Figure S9: A histogram of H−Cl bond lengths seen in universal potentials (a) and fine-tuned potentials (b). Panel (a) depicts H−Cl bond lengths from two trajectories generated by a universal potential. Panel (b) depicts H−Cl bond lengths from a universal potential (U) and a fine-tuned potential (FT1)

Cobalt Analysis

We tie a change in regime to a change in the coordination environment of cobalt that occurs 6 ns into production dynamics sampled by N-50pts. Table 4 reveals that the naive

model decreases in error from 12.7 meV/at to 7.99 meV/at for frames before and after 6 ns, respectively. We are then interested in observing how this change is reflected in PC space with respect to each coordination environment. Figure S10 illustrates the change in coordination environment, and its effects through PCA and Q-residual analysis.

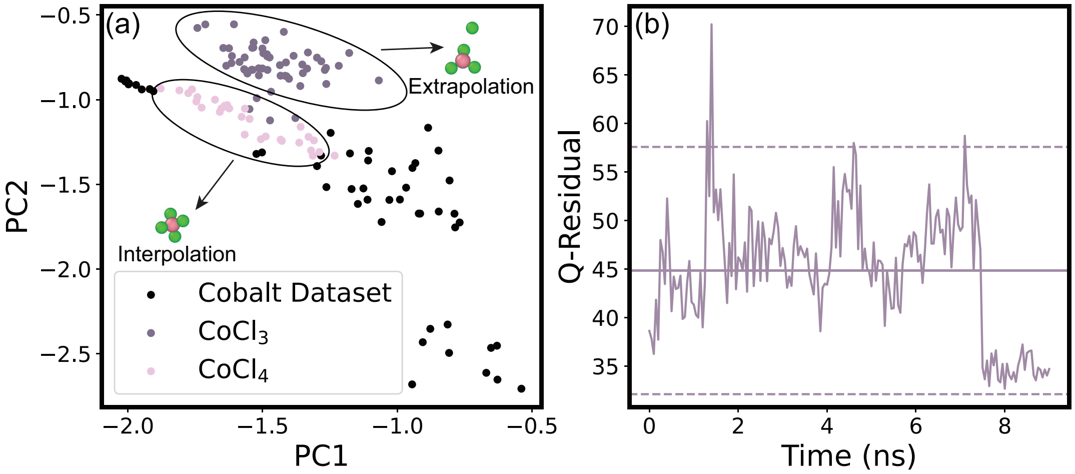

Figure S10: A PCA/Q-residual analysis on cobalt environments. The left panel (a) separates the trajectory sampled by N-50pts into images taken before 6 ns, and after 6 ns, where cobalt exists as either $CoCl_3$ or $CoCl_4$, and the images of cobalt in N-50pts fine-tuning dataset. We label the two regimes as interpolative and extrapolative based on visual inspection of the PCA. Lavender points represent environments where cobalt exists as $CoCl_3$, pink $CoCl_4$, and black is the dataset. The right panel (b) represents the Q-residual of cobalt over the length of the sampled trajectory, with the mean and $\pm$ 2 standard deviations plotted as horizontal lines.

Figure S10 illustrates a similar analysis as Figure 8 and Figure 9. Here, the Q-residual is displayed on the right panel, and the left panel displays the PCA of the cobalt environments encountered in the 9 ns trajectory from N-50pts, and the dataset of cobalt environments used to train N-50pts. We divide the trajectory into before and after 6 ns, as we see different accuracies shown in Table 4. We label the region where cobalt exists as $CoCl_3$ as an extrapolative region, and $CoCl_4$ as an interpolative region, as these points can be directly interpolated from the dataset in PC1 and PC2, and the extrapolative region cannot.

Unfortunately, there does not appear to be a direct mapping from Q-residuals to the artifacting in cobalt, highlighting another limitations of this method of outlier detection.

We believe that the lack of a trend in Q-residuals is due to insufficient data, as there are only 50 cobalt environments avaliable in the reference dataset.

While the Q-residuals do not present a clear pattern, we attribute the shift in accuracy presented in Table 4 to the shift in cobalt coordination. We divide the trajectory into two regions based on the findings in Figure 5a, corresponding to extrapolative and interpolative behavior. Environments before 6 ns are labeled as extrapolative, while those after 6 ns are labeled as interpolative. In the extrapolative region, environments correspond to $CoCl_3$, whereas in the interpolative region they correspond to $CoCl_4$. After 6 ns, the shifts cobalt into a region where direct interpolation of the reference dataset is possible, as evidenced by the PCA. Consistently, N-50pts is more accurate after 6 ns, supporting the idea that interpolation is taking place. As different chemical environments in just cobalt lead to shifts in aaccuracy this raises the question of how accurate the dynamics are while extrapolating. Furthermore, when we evaluated these differing chemical environments using FT5 in Table 4, FT5 remains consistently more accurate than N-50pts.

Dilute LiCl + CoCl2 System

We perform the iterative and naive workflows for a simple lithium chloride and cobalt chloride system dissolved in water. The system contains 55 water atoms, one lithium (I) chloride, and one cobalt (II) chloride. This demonstrates the limitations of these models on simpler systems and represents a systematic bias throughout different chemistries for liquid simulations. Figure S11 describes the results when each model samples a 1 ns MD trajectory as a residual against DFT.

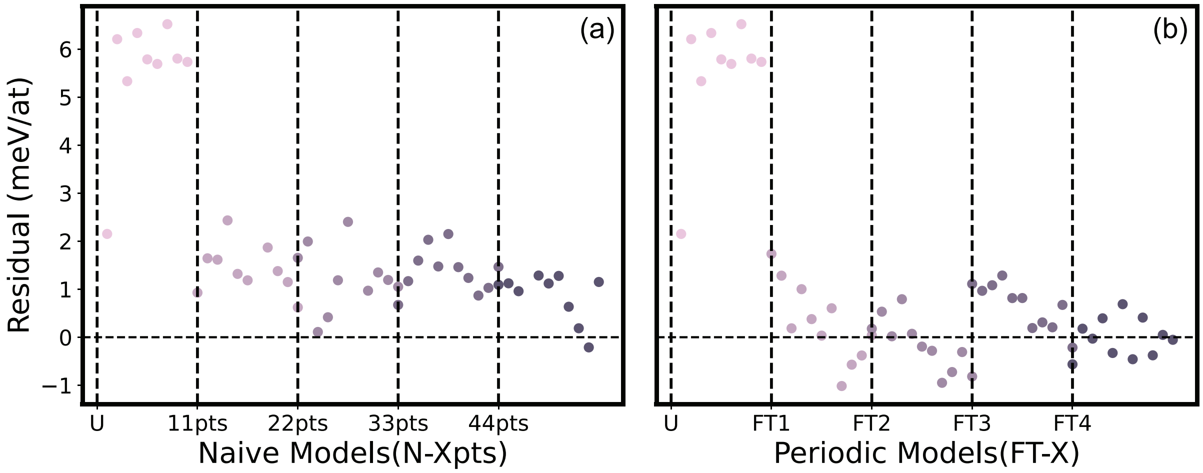

Figure S11: The naive and iterative workflows expanded to a system of dilute lithium (I) chloride and cobalt (II) chloride. We allow each model to sample a 1 ns trajectory, with the naive workflow demonstrated on the left panel (a), and the iterative workflow demonstrated on the right panel (b).

The data distribution of our iterative dataset is lower than the naïve dataset, and further iterations tend to get more stable structures. This again indicates that there is an offset due to bias that limits the naive trajectory from learning structures relevant to dynamics.

Atomic Force Parity Plots

Each of the following parity plots is taken from the 9 ns trajectory (Figure 5), comparing MLIP evaluations versus DFT evaluations.

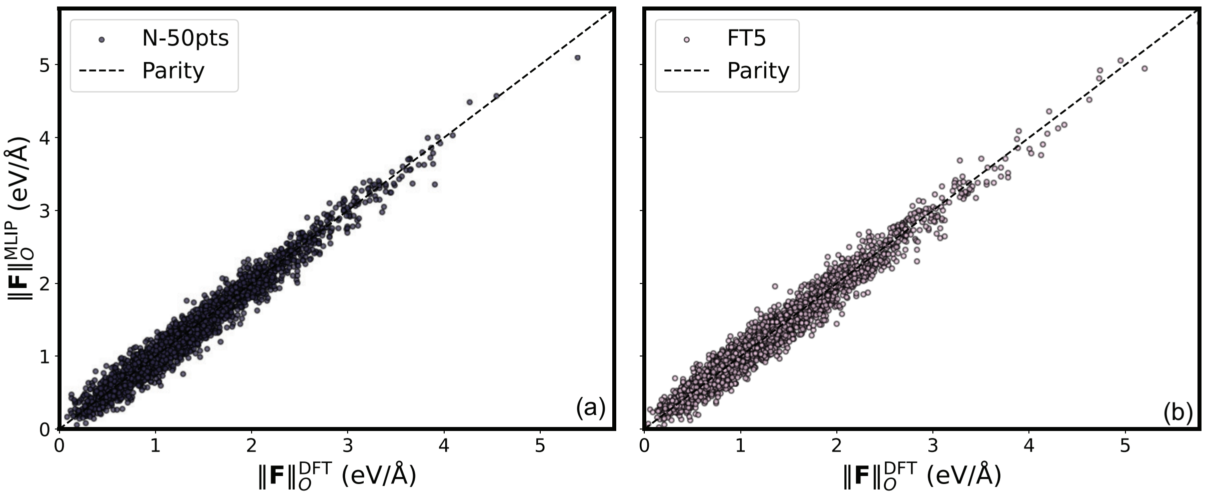

Figure S12: Parity plot of O force magnitudes of sampled O environments along a 9 ns trajectory for (a) a naively trained model and (b) an iteratively trained model.

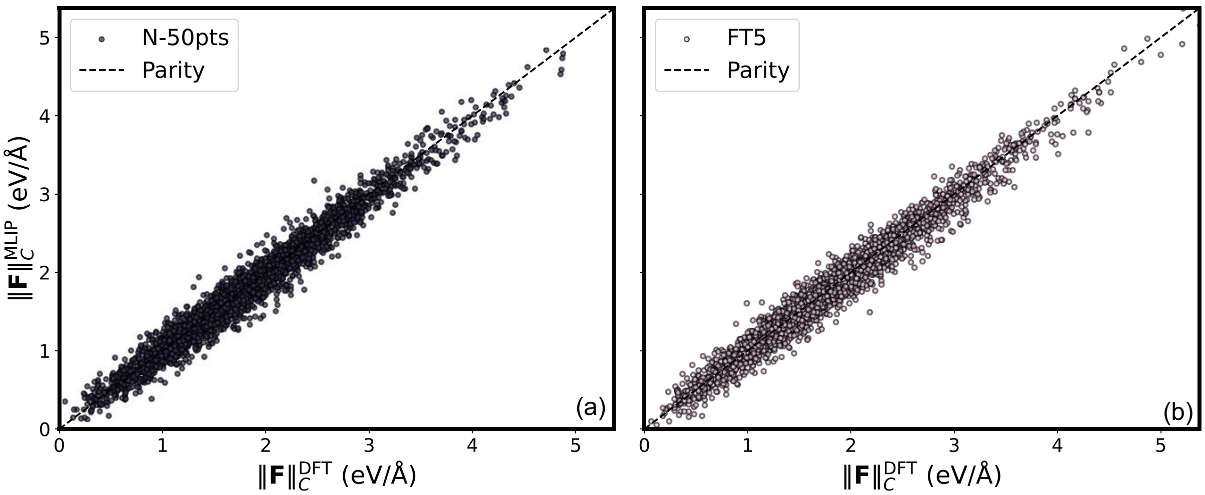

Figure S13: Parity plot of C force magnitudes of sampled C environments along a 9 ns trajectory for (a) a naively trained model and (b) an iteratively trained model.

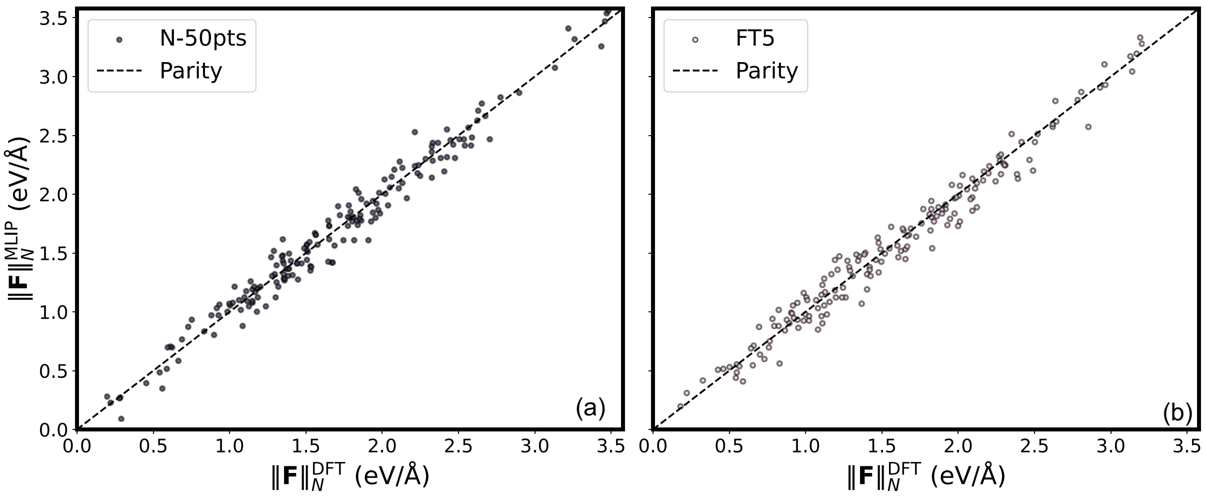

Figure S14: Parity plot of N force magnitudes of sampled N environments along a 9 ns trajectory for (a) a naively trained model and (b) an iteratively trained model.

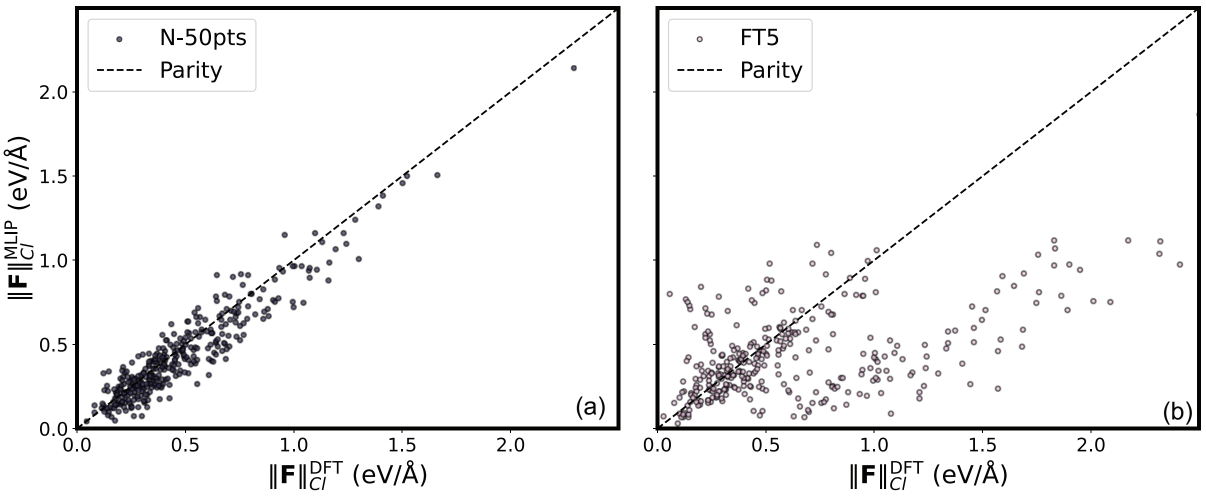

Figure S15: Parity plot of Cl force magnitudes of sampled Cl environments along a 9 ns trajectory for (a) a naively trained model and (b) an iteratively trained model.

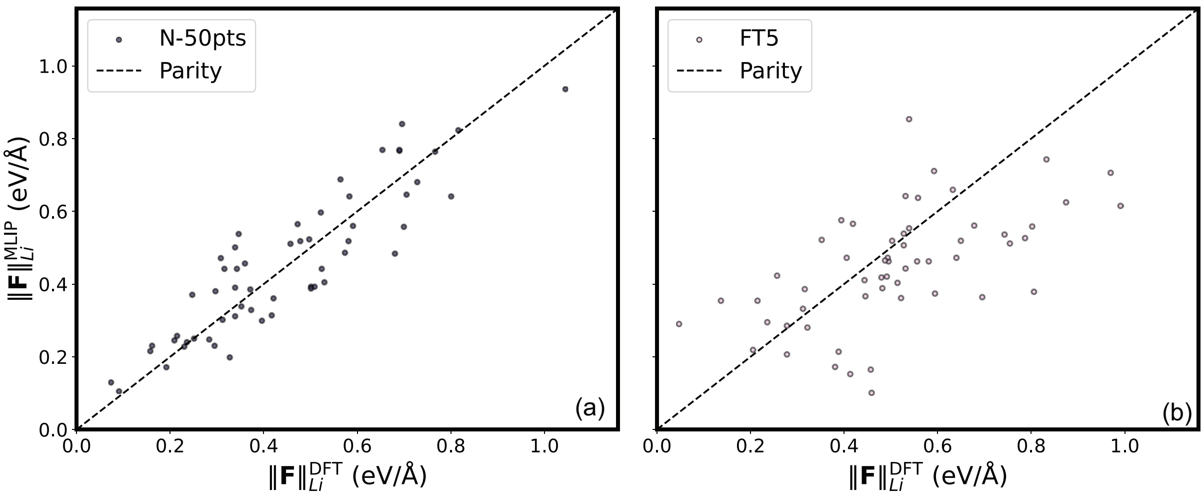

Figure S16: Parity plot of Li force magnitudes of sampled Li environments along a 9 ns trajectory for (a) a naively trained model and (b) an iteratively trained model.

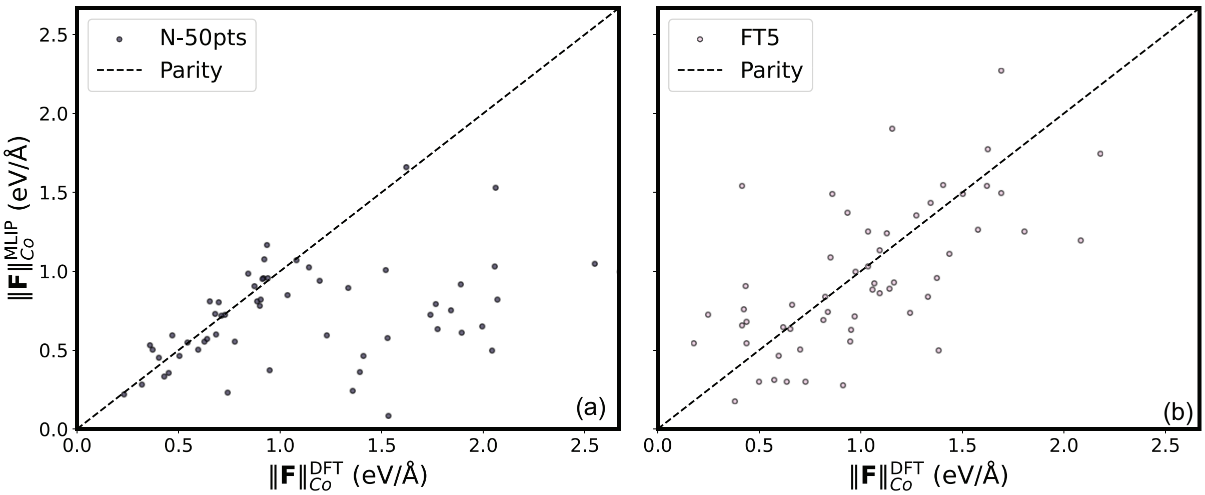

Figure S17: Parity plot of Co force magnitudes of sampled Co environments along a 9 ns trajectory for (a) a naively trained model and (b) an iteratively trained model.

We further decompose these parity plots into relative force errors, normalized by the mean force in Table S3

Table S3: Per-element force errors for FT5 and N-50pts evaluated on their respective 9 ns trajectories. The relative force error is computed as RMSE_F / Mean $|F_\alpha|$, where the mean refers to the average force component for that element in the trajectory.

	FT5 N=56			N-50pts N=58		
Element	RMSE_F eV/Å	Mean $\lvert F_\alpha \rvert$ eV/Å	Rel. F Err %	RMSE_F eV/Å	Mean $\lvert F_\alpha \rvert$ eV/Å	Rel. F Err %
C, N=51	0.132	0.903	14.6	0.142	0.894	15.9
N, N=3	0.111	0.798	13.9	0.113	0.828	13.6
O, N=45	0.122	0.666	18.3	0.117	0.674	17.4
H, N=90	0.060	0.460	13.0	0.073	0.457	16.0
Cl, N=6	0.331	0.332	99.7	0.092	0.241	38.2
Li, N=1	0.173	0.252	68.6	0.080	0.230	34.8
Co, N=1	0.382	0.493	77.5	0.543	0.562	96.6

Table S3 shows the force errors per atom type, and the mean-normalized relative force errors presented in Figure S17, S15, S16, S13, S14, S12, and Figure 7. We observe comparable relative errors of about 15% for C, N, O, and H, which make up the bulk solvent, indicating low force errors on the bulk dynamics. However, the solvated ions, Cl, Co, and Li have larger errors, which span from 35% to 100%. Average errors capture the typical error levels, but do not capture the distributions and outliers. We further decompose the errors into error distributions via violin plots in Figure S18, which shows the force error distributions by atom type.

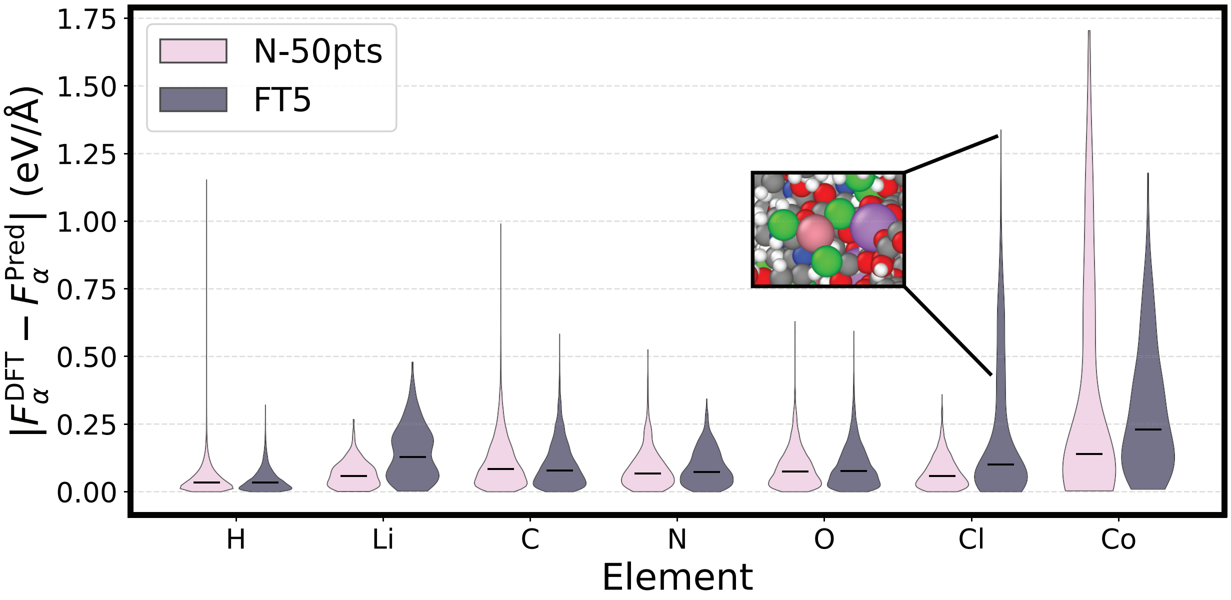

Figure S18: Violin plot of the force error distribution for the 8 ns simulations sampled by the naive and iterative workflow. The inlay represents the chlorine environment that yields the highest errors, which is an unphysical $LiCoCl_3$ coordination environment (Li: pink, Cl: green, Co: purple).

Figure S18 shows the force error distributions of each atom type. The plots for Li and Co are challenging to interpret because there are only about 60 frames, or 180 force components of each atom. However, Cl is interesting because it is predicted considerably worse in FT5 compared to in N-50pts (Table S3). We attribute this misprediction to the absence of $LiCoCl_3$ in the FT5 dataset. This apparent misprediction further motivates other fine-tuning approaches that integrate more diversity through multiple starting configurations.

Another interesting observation from Figure S18 is the fact that N-50pts has a significantly higher variance, which is consistent with the model artifacts that are observed in its MD trajectory. We quantify these distributions further in Table S4, where we present the mean, median, 95th and 99th percentile, and the max force error.

Table S4: Per-element distributions of force-component absolute errors $|F_\alpha^{\mathrm{DFT}} - F_\alpha^{\mathrm{Pred}}|$ for FT5 and N-50pts, evaluated on each model's own 9 ns trajectory. The α subscript represents Cartesian components (x, y, z). All values in eV/Å.

	FT5					N-50pts				
Element	Mean	Median	95th	99th	Max	Mean	Median	95th	99th	Max
C	0.101	0.079	0.264	0.370	0.583	0.108	0.085	0.286	0.404	0.990
N	0.090	0.073	0.206	0.283	0.343	0.086	0.068	0.231	0.338	0.525
O	0.095	0.076	0.245	0.339	0.594	0.090	0.074	0.235	0.322	0.629
H	0.045	0.033	0.127	0.183	0.321	0.049	0.035	0.134	0.223	1.153
Cl	0.216	0.101	0.747	0.974	1.337	0.071	0.058	0.181	0.250	0.359
Li	0.142	0.129	0.319	0.386	0.479	0.065	0.058	0.142	0.212	0.267
Co	0.299	0.230	0.752	0.967	1.178	0.347	0.139	1.218	1.586	1.705

These analyses show that, aligning with our original tables, the mean force errors are essentially equivalent for C, N, O, and H, which comprise of most of the system. However, N-50pts shows notably higher variance for hydrogen, with max errors of 1.15 eV/Å, as compared to FT5's max error of 0.32 eV/Å. This aligns with our observations that the failure mode of N-50pts is localized to environments outside of the fine-tuning configurational space, and manifests through high-force errors, which we demonstrate through our analysis with Q-residuals (Figure 8, 9) and parity plots (Figure 7).

TICA Analysis

We perform a time-lagged independent component analysis (TICA)[4] on the long trajectories to evaluate the long-term stability of the fine-tuned models. We maintain the same SOAP hyperparameters as the main text, and compile SOAP descriptors every 5 ps, resulting in 1,801 frames. To make the calculation computationally feasible, we reduce the dimension of SOAP vectors using a 10-component PCA to fit the SOAP descriptors, capturing 92.6% and 92.7% of the variance for the naive and iterative trajectories, respectively. We then fit a TICA model using the `deeptime` python package[5] with a lagtime $\tau = 10$ frames to the PCA vectors and plot the first two dimensions in Figure S19

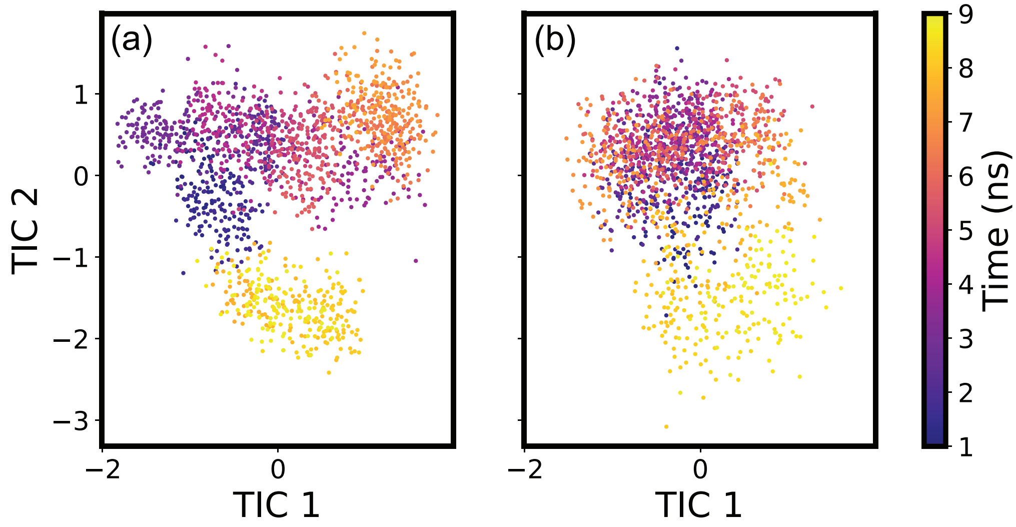

Figure S19: Time-lagged Independent Component Analysis (TICA) dimensionality reduction for the trajectories sampled by (a) N-50pts and (b) FT5.

As a baseline, we also include the TICA analysis of a 4.4 ns simulation from a universal potential.

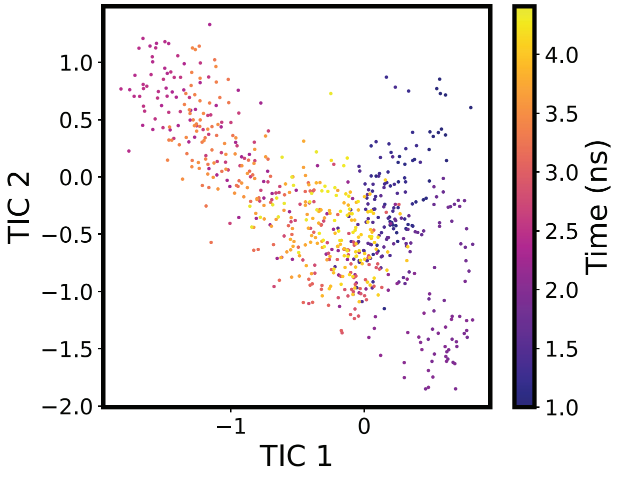

Figure S20: Time-lagged Independent Component Analysis (TICA) dimensionality reduction for a 4.4 ns simulation from the universal potential.

Figures S20 and S19 show the TICA decomposition of production trajectories from the universal potential, as well as the fine-tuned potentials N-50pts and FT5, respectively. For comparison, the universal appears to move linearly along the TICA dimensions, representing

a slow dynamic mode throughout the trajectory. Similarly, the 8 ns trajectory sampled by N-50pts also exhibits systematic drift. In contrast, the trajectory sampled by FT5 exhibits a noisy TICA decomposition, which indicates that the trajectory is largely capturing thermal noise, with little long-term movement over the trajectory. These results indicate that the trajectories sampled by the uMLIP and N-50pts both exhibit movement outside thermal noise, which is unexpected for the system.

References

(1) Cococcioni, M.; de Gironcoli, S. Linear response approach to the calculation of the effective interaction parameters in the LDA+U method. *Physical Review B* **2005**, *71*, 035105.

(2) Jain, A.; Hautier, G.; Moore, C. J.; Ping Ong, S.; Fischer, C. C.; Mueller, T.; Persson, K. A.; Ceder, G. A high-throughput infrastructure for density functional theory calculations. *Computational Materials Science* **2011**, *50*, 2295–2310.

(3) Moore, G. C.; Horton, M. K.; Ganose, A. M.; Siron, M.; Linscott, E.; O'Regan, D. D.; Persson, K. A. High-throughput determination of Hubbard U and Hund J values for transition metal oxides via linear response formalism. *Physical Review Materials* **2024**, *8*, 014409.

(4) Molgedey, L.; Schuster, H. G. Separation of a mixture of independent signals using time delayed correlations. *Physical Review Letters* **1994**, *72*, 3634–3637.

(5) Hoffmann, M.; Scherer, M.; Hempel, T.; Mardt, A.; de Silva, B.; Husic, B. E.; Klus, S.; Wu, H.; Kutz, N.; Brunton, S. L.; Noé, F. Deeptime: a Python library for machine learning dynamical models from time series data. *Machine Learning: Science and Technology* **2021**, *3*, 015009.
